# Immunomodulation of COVID‐19 severity by helminth co‐infection: Implications for COVID‐19 vaccine efficacy

**DOI:** 10.1002/iid3.573

**Published:** 2021-12-03

**Authors:** Yibeltal Akelew, Henok Andualem, Endris Ebrahim, Aytenew Atnaf, Wasihun Hailemichael

**Affiliations:** ^1^ Immunology and Molecular Biology, Medical Laboratory Sciences, College of Health Sciences Debre Markos University Debre Markos Ethiopia; ^2^ Immunology and Molecular Biology, Medical Laboratory Sciences, College of Health Sciences Debre Tabor University Debre Tabor Ethiopia; ^3^ Immunology and Molecular Biology, Medical Laboratory Sciences, College of Health Sciences Wollo University Dessie Ethiopia; ^4^ Hematology and Immunohematology, Medical Laboratory Sciences, College of Health Sciences Debre Markos University Debre Markos Ethiopia

**Keywords:** COVID‐19, helminth, immunomodulation, SARS‐CoV‐2, vaccine

## Abstract

Severe acute respiratory syndrome coronavirus 2 (SARS‐CoV‐2), an emerging virus in late 2019 causing coronavirus disease 2019 (COVID‐19), has caused a catastrophic effect, resulting in an unprecedented global crisis. The immunopathology of COVID‐19 appears to be clearly associated with a dysregulated immune response leading to organ failure and death. Similarly, over two billion people worldwide are infected with helminth, with those living in low‐middle‐income countries disproportionately affected. Helminth infections have been shown to possess immunomodulatory effects in several conditions. Helminth co‐infection in COVID‐19 patients is one of the potential reasons for global attention to answer why COVID‐19 severity is still lower in helminth endemic countries. Recent studies have shown that helminth endemic countries showed fewer cases and deaths so far and helminth co‐infection might reduce the severity of COVID‐19. Moreover, lessons from other diseases with helminth co‐infection have been shown to substantially reduce vaccine efficacy that could also be implicated for COVID‐19. This immunomodulatory effect of helminth has intended and unintended consequences, both advantageous and disadvantageous which could decrease the severity of COVID‐19 and COVID‐19 vaccine efficacy respectively. Herewith, we discuss the overview of COVID‐19 immune response, immunomodulatory effects of helminth co‐infections in COVID‐19, lessons from other diseases, and perspectives on the efficacy of COVID‐19 vaccines.

## Graphical abstract

Helminth co‐infection could modulate the severity of COVID‐19. Not all parasites will have immunomodulatory properties. Lessons from respiratory viruses coinfected with helminth have been shown reduced vaccine efficacy. Helminth co‐infection and antihelminthic therapy could reduce and enhance the efficacy of COVID‐19 vaccines, respectively.

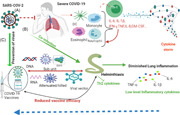

## 1 INTRODUCTION

Since its first outbreak in late November 2019 from Wuhan, Hubei Province, China, the infection of coronavirus, SARS‐CoV‐2, an etiology of COVID‐19 has engendered unprecedented global crisis.^1^ As of October 27, 2021, more than 244 million and 4.96 million people were infected and died globally.^2^ As a result, scientists all over the world have made tremendous efforts to produce different vaccines and antiviral drugs. Now, multiple COVID‐19 vaccines have been successfully developed much faster than the development of any other vaccines. SARS‐CoV‐2 is a single‐stranded RNA virus (ssRNA) where the structural protein includes a Spike (S), Membrane (M), Envelope (E), and Nucleocapsid (N).^3^ The spike (S) protein serves for the binding of SARS‐CoV‐2 to its receptor human angiotensin‐converting enzyme 2 (ACE2) through its receptor‐binding domain and further promotion of virus entry in to the cell‐mediated by human TM protease serine 2 (TMPRSS2) which has been found to co‐express, co‐localize and interact with ACE2.^4^, ^5^ Following receptor binding, the virus can enter the cell cytoplasm via clathrin‐mediated endocytosis.^6^

### 1.1 Immune responses to SARS‐CoV‐2

An effective immunologic response against SARS‐CoV‐2 requires both wings of immunity. Innate recognition of virus by pattern recognition receptor (PRR) like toll‐like receptors (TLRs) and retinoic acid‐inducible gene (RIG‐1) results in the activation of the transcription factors, nuclear factor kappa‐light‐chain‐enhancer of activated B cells (NF‐kB), and interferon regulatory factor 3 (IRF3), resulting in translocation into the nucleus and inducing the expression of pro‐inflammatory cytokines, chemokines, and type I interferon (IFN).^7^ Besides upregulation of type 1 IFN gene and induction of aberrant inflammatory cytokines and chemokine's secretion like interleukin (IL)‐6, IL‐1β, IL‐8, CCL2, CCL8, and CXCL9, exuberant activation of complement pathways results the overproduction of chemoattractants, C3a and C5a (anaphylatoxins) which will further induce recruitment of inflammatory cells leading cytokine storm.^8^

Adaptive immune response against SARS‐COV‐2 has been revealed a positive association between antibody response and T‐cell immune memory with disease severity.^9^ Severe COVID‐19 can also result CD4^+^ and CD8^+^ T cells exhaustion with increased cell surface expression of programmed cell death protein 1 (PD‐1) and T‐cells immunoglobulin and mucin domain 3 (Tim‐3).^10^

While the pathology of SARS‐CoV‐2 is not equivocally understood, at least we know it is mainly associated with hyperinflammatory responses, as characterized in the severe cases of patients. SARS‐COV‐2 dampens the antiviral IFN responses through unrestrained virus replication in several cells which results in the upregulation of activated macrophages, neutrophils, and other adaptive immune cells which lead to elevated pro‐inflammatory cytokines such as IL‐1β, IL‐6, and TNF‐α.^11^, ^12^ The reduced innate antiviral defenses coupled with hyperproduction of inflammatory mediators are the determinant factors of severe COVID‐19.^13^

Several studies have revealed that severe COVID‐19 disease is characterized by increased levels of inflammatory cytokines, and chemokines as shown in Table 1.

**Table 1:** The immune response profiles in severe SARS‐CoV‐2 infected patients

Authors	Country of study	Year	Study design with no. of study subjects (*n*)	Immune signature linked to severe COVID‐19	Refs.
Chen et al.	China	2020	Retrospective (*n* = 21)	Higher levels of IL‐2R, IL‐6, IL‐10, and TNF‐α and lower IFN‐γ production by CD4^+^ and CD8^+^ T and NK cell	^14^
			Prospective (*n* = 29)	Higher levels of IL‐2R and IL‐6	^15^
			Retrospective (*n* = 48)	Elevated IL‐6	^16^
			Retrospective (*n* = 548)	Elevated IL‐6 and Decreased lymphocytes, CD8^+^ T‐cell, eosinophils, and platelets, Increased neutrophil count and neutrophils‐to‐lymphocytes ratio	^17^
Chi et al	China	2020	Prospective (*n* = 70)	Higher levels of IL‐6, IL‐7, IL‐10, IL‐18, G‐CSF, M‐CSF, MCP‐1, MCP‐3, IP‐10, MIG, and MIP‐1α	^18^
Del Valle et al	USA	2020	Cohort (*n* = 231)	Higher levels of IL‐6, IL‐8, and TNF‐α	^19^
Han et al.	China	2020	Prospective (*n* = 102)	Higher levels of IL‐6, CRP, and IL‐10	^20^
Herold et al.	Germany	2020	Cohort (*n* = 89)	Elevated IL‐6 and CRP	^21^
Huang et al.	China	2020	Prospective (*n* = 41)	Higher plasma levels of IL2, IL7, IL10, G‐CSF, IP10, MCP1, MIP1A, and TNF‐α	^22^
Luo et al.	China	2020	Retrospective (*n* = 1018)	Elevated IL 6 and lower CD8^+^ T cell counts	^23^
McElvaney et al.	Ireland	2020	Longitudinal cohort (*n* = 70)	Higher levels of IL‐1 β, IL‐6, and sTNFR1	^24^
Merza et al.	Iraq	2020	Prospective (*n* = 128)	Higher IL‐6, IL‐8, and IL‐10 and lower IFN‐γ and IL‐4	^25^
Tan et al.	China	2020	Retrospective (*n* = 90)	Lymphopenia	^26^
Wan et al.	China	2020,	Longitudinal (*n* = 123)	Higher levels of CD4^+^ T, CD8^+^ T, IL‐6, and 10	^27^
Yang et al.	China	2020	Prospective (*n* = 50)	IP‐10, MCP‐3, HGF‐α, MIG, MIP‐1α, and IL‐1Rα	^28^

Abbreviations: CD8+, cytotoxic T cell; COVID‐19, coronavirus disease; CRP, C‐reactive protein; G‐CSF, granulocyte colony‐stimulating factor; HGF, hepatocyte growth factor; IFN‐γ, interferon‐gamma; IL‐1β, interleukin‐1β; IL‐2R, interleukin‐2 receptor; IP‐10, IFN‐γ inducible protein‐10; MCP‐1, monocyte chemoattractant protein‐1; M‐CSF, macrophage colony‐stimulating factor; MIG, monokine induced by interferon‐γ; MIP‐1α, macrophage inflammatory protein‐1 alpha; NK cell, Natural killer cells; sTNFR1, soluble tumor necrosis factor receptor 1; TNF‐α, tumor necrosis factor α.

**Figure 1:**
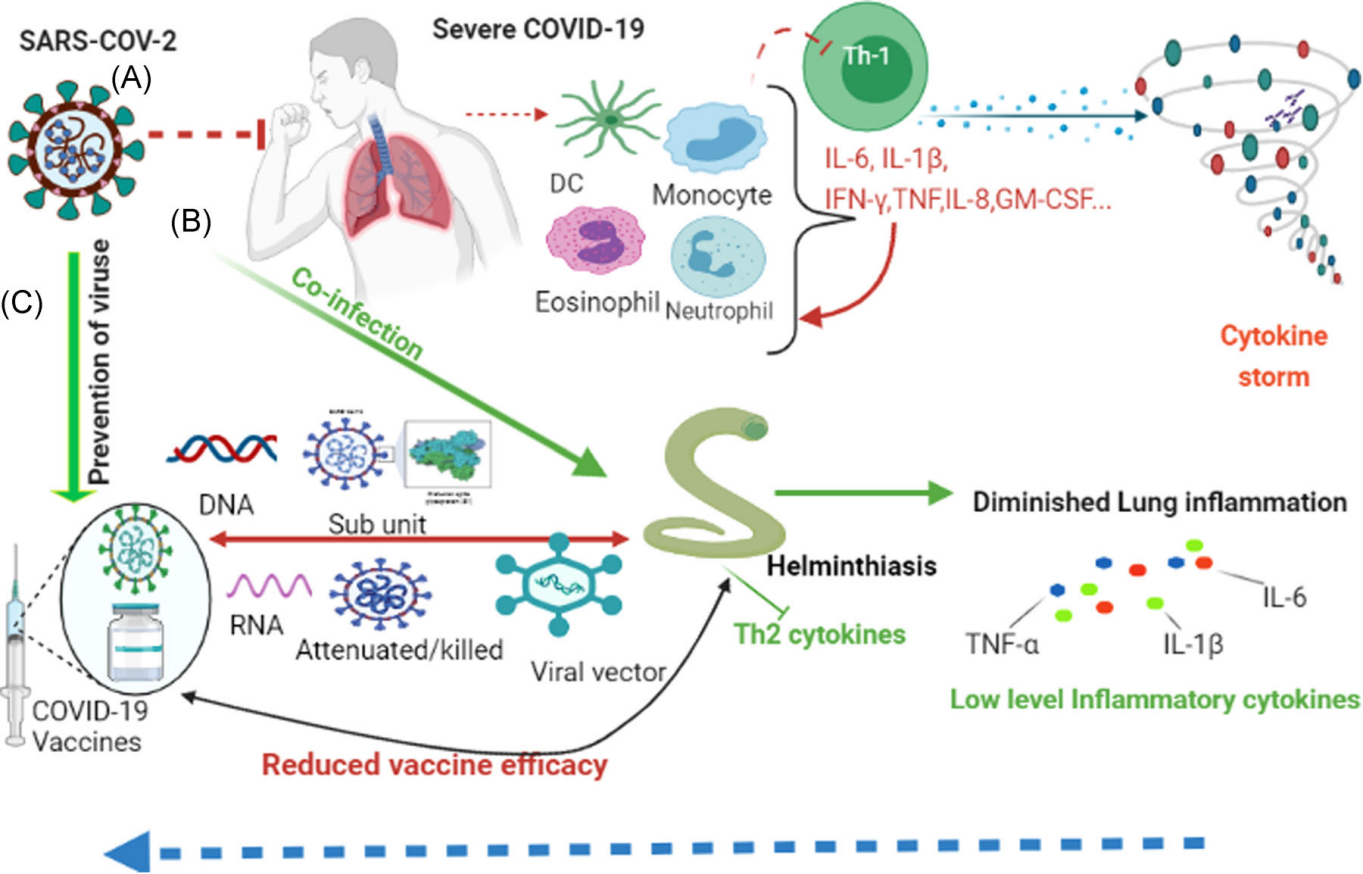
(A) illustrates the immune response in severe COVID‐19 characterized by activation of pro‐inflammatory cytokines mediated by TH1 cells leading to cytokine storm. (B) shows the immunomodulatory effects of helminth co‐infection in COVID‐19 patients. COVID‐19 patients have reduced TH1 response due to Th2 mediated helminth immune response. (C) shows the different available vaccine efficacy has not been assessed so far. Probably it could reduce the efficacy of the COVID‐19 vaccine based on lessons from other diseases including respiratory viruses. The figure is created with https://app.biorender.com

These cytokines and chemokines in turn attract other immune cells to migrate to the site of inflammation thereby cascading the intensification of inflammatory response as shown in Figure 1. It has been reported that Th2/Th1 cytokine imbalance is related to higher risk of mortality.^29^ Hence, the presence of excessive production of pro‐inflammatory cytokines, referred to as “cytokine storm”; which leads to widespread tissue damage involving acute respiratory distress syndrome or multiorgan failure which is linked with mortality in COVID‐19 patients.^10^, ^30^ As a result, immunomodulatory therapeutic approaches targeting the pro‐inflammatory cytokines have been applied to alleviate COVID‐19 severity.^31^, ^32^ Keep this in mind, herewith; we elucidate the interaction of helminth with other diseases, and insights to SARS‐COV‐2 and the potential implications of helminth co‐infection in the context of COVID‐19 vaccine efficacy.

## 2 IMMUNOMODULATORY EFFECTS OF HELMINTH; THE ERA OF SARS‐COV‐2 INFECTION

Parasitic helminths are still a major health problem worldwide, affecting more than 2 billion people (approximately one‐third of mankind) where most helminth infections are observed among the resource‐limited countries.^33^ According to the World Health Organization (WHO) report, sub‐Saharan Africa accounts 85% of the neglected tropical disease burden resulting from helminth infections.^34^

Helminth infections are known to induce immune regulatory responses in the host that can be helpful to control inflammation.^35^ In paradox, helminth infections do have a significant public health burden, particularly in low and middle‐income countries,^36^, ^37^, ^38^ associated with high rates of morbidity, with chronic infection typically resulting in anemia and malnourishment and even severe manifestations like elephantiasis and blindness.^39^, ^40^ Helminth‐induced immunomodulation reduces the host immune response by permitting parasite survival and minimizes tissue damages. As a result, most peoples with helminths are unaware of their infection.

### 2.1 Modulation of the innate immune response by helminth

Innate immunity is the first line response and typically identifies pathogens based on germline‐encoded receptors that detect the conserved components of pathogens.

Helminths and productions of excretory/secretory product are recognized by receptors of phagocytes and other cells types, which participate in an intricate cytokine network to generate innate Th2 responses which in turn drives the polarization of AAM, and the activation of eosinophils, basophils, innate lymphocyte T cells 2 (ILC2s), and mast cells.^41^, ^42^ The fact that helminth parasites negatively regulate TLRs to a much larger degree suggests that the immune response to helminths could also influence subsequent activation of B and T lymphocytes.^43^ Besides TLR signaling modulation, helminth‐derived immunomodulatory molecules such as cytokine & innate defense homologs and growth factors, enzymes and inhibitors, lipids, and lipid‐binding mediators have been largely revealed in modulation of the innate immune response.^44^

### 2.2 Modulation of the adaptive immune response by helminth

It is clear that adaptive immunity is initiated when an innate immune response fails to eliminate a pathogen. The infection with helminth modulates CD4^+^ T cell differentiation, induces the activity of regulatory T cell (Treg) responses, regulates immunoglobulin class switching, and induces a regulatory B cell (Breg) response, and thereby dampening the host immune responses.^44^ These complex interactions result in the skewness of host immunity towards the type 2 immune response.^45^ This is typically characterized by the induction of cytokines such as IL‐4, IL‐5, IL‐9, IL‐13, and IL‐21, and usually the absence of IFN‐γ and IL‐17 production.^39^ Besides, the Th2 responses support the initiation of Treg response that dampens the host Type 1 immune response pathway^45^, ^46^ (Table 2). Many helminth species secrete a plethora of immunomodulatory proteins that bind to cellular receptors and induce the production of IL‐10 from cellular sources. These proteins may also block chemokine release, and Treg development, or inhibit B cell regulatory signaling and transendothelial migration.^44^

**Table 2:** An overview of studies focusing on the immune response profiles of helminth infections

Author	Helminth species	Year, study subjects, and country of study	Methodology	Major findings	Refs.
Figueiredo et al.	*T. trichiura* and *A. lumbricoides*	2010, Brazil children aged to 4–11 (*N* = 1060)	Cytokine measurement using unstimulated and stimulated with mitogen or *A. lumbricoides* antigens	Th2 immune response (increased production IL‐10, IL‐5, and IL‐13)	^47^
Shalaby and Shalab	Ascarislumbricoides	2016, Egypt (*N* = 60) 7−15 years children	Serum cytokine profile using ELISA	High levels of IL‐4 and IL‐5	^48^
Ferreira et al.	*Ancylostoma caninum* excretory/secretory products (AcES)	2013, Australia	AcES suppress intestinal pathology pro‐inflammatory cytokine expression in mice model of colitis	Type 2 cytokine response (increased IL‐4, IL‐10, AAM, and eosinophils, downregulation of pro‐inflammatory cytokines)	^49^
Doyen et al.	Hookworm	2021, Belgium N = 20 cases and 14 controls	Evaluation of serum cytokine before and after Hookworm treatment	A decrease in Treg which exhibited a decrease parallel to Th2 response	^50^
Kron et al.	*Brugia malayi*	2013, USA	Asparaginyl‐tRNA synthetase E/S protein in mice model of colitis	Resolves intestinal inflammation, induced regulatory responses and IL‐10 in mice with T‐Cell transfer colitis	^51^
Ferreira et al.	Hookworm	2017, Australia	Hookworm recombinant AIP‐1 in mice model of colitis	Increased IL‐10, TGF‐β, and TSLP resulted in the suppression of TNF‐α, IL‐13, and IL‐17 A and GM‐CSF, CXCL‐11, and COX‐2 mRNA transcripts	^52^
Sanin and Mountford	*S. mansoni*	2015UK	*S. mansoni* cercarial Sm16 E/S in murine model	Blocks classical activation of macrophages to LPS or IFN‐γ. Induces IL‐10, Inhibit IL‐12p40 and macrophage activation in response to TLR4 and TLR3 TLR ligands. production	^53^

Abbreviations: (COX)‐2, cyclooxygenase; GM‐CSF, granulocyte‐macrophage colony‐stimulating factor; (TNF)‐α, tumor necrosis factor; AAM, alternatively activated macrophage; AIP‐1, anti‐inflammatory protein‐1; CXCL, CX motif chemokine; E/S, excretory/secretory; ELISA, enzyme‐linked immunosorbent assay; IL‐10, interleukin‐ 10; LPS, Lipopolysaccharide; TGF‐β, transforming growth factor; TLR, toll‐like receptor; TSLP, thymic stromal lymphopoietin.

### 2.3 Immunomodulation of helminth during co‐infections

Helminth infections are notable to modulate systemic pro‐inflammatory cytokines and chemokines, which show a significant implication in a wide range of comorbidities. For example, a study done in India among type 2 diabetes mellitus patients shows that *Strongyloides stercoralis* alleviated the pro‐inflammatory milieu while anthelmintic therapy partially restores the plasma pro‐inflammatory cytokine and chemokine levels.^54^ Helminth immunomodulatory effects have been also observed in many other conditions such as *Mycobacterium tuberculosis* infection,^55^, ^56^ atopy, asthma,^57^ and Autoimmune disease.^58^ Besides this, helminth secretome has recently been shown as a novel therapeutic avenue for inflammatory disorder.^59^ Helminth or its derived product treatment induces Treg and/or alternatively activated macrophages (AAMs) which could directly slow down allergen‐specific Th2 responses through a cell contact‐dependent mechanism, synthesis of common immunomodulatory mediators such as IL‐10 and TGF‐ß inhibiting IFN‐γ secreting cell (Th1), and inhibition of binding allergen‐specific IgE via helminth‐specific, and nonspecific polyclonal IgE production called IgE blocking hypothesis.^60^

The hypothesis that helminths infection modulates the immune response to viral infection is evident and corroborated by an experimental study by Rolot et al. The study uses the inoculation of eggs and adults of *Schistosoma mansoni* to murid herpesvirus 4 (MuHV‐4) infected mice. They explored the helminths induced IL‐4 dependent (possibly Th2 source) control of virus.^61^ Similar study has also shown that the helminth‐derived immunomodulator AvCystatin, derived from filarial nematode reduced *respiratory syncytial virus* (RSV) associated inflammations by inducing CD4^+^ T cells producing IL‐10 cytokine.^62^ In addition, helminths such as *Heligmosomoides polygyrus* can induce a protective antiviral response to respiratory syncytial virus. This is primarily mediated through the interaction between microbiota and upregulation of type‐I IFN signaling.^63^ On the other hand, a study by Osborne et al.^64^ showed co‐infection of helminth in viral infection resulted in diminished antiviral immunity, which is highly dependent on Ym1, a chitinase‐like molecule that was associated with AAM without changes in the microbiota. However, the antiviral immunity was partly restored by the neutralization of Ym1.^64^ Such scenarios could have a similar impact on immunity to COVID‐19 aforementioned and which in turn affect the response to the COVID‐19 vaccine.

After the emergence of SARS‐COV‐2, several reports on the severity of COVID‐19 variations among different countries with the possible factors have been raised from researchers all over the world. The observation that the low number of severe cases and deaths due to COVID‐19 in resource‐limited nations has been a puzzle for scientists.

The situation was similar in settings where there is a high Bacillus Calmette–Guérin (BCG) vaccine coverage and helminth infections, which has attention. This was the concept of trained immunity that defines the innate immune response to induce memory more specifically BCG could result in protection against SARS‐CoV‐2 infection and might reduce the severity of COVID‐19.^65^, ^66^, ^67^ Moreover, based on the recent data reviewed, the high reactivity of BCG‐derived antigen to its corresponding SARS‐COV‐2 substantially increased type II IFN production and its effect on CD4^+^ T‐cells and nonspecific immune responses could harness cross‐protection against severe COVID‐19.^66^

Despite, studies not explored and compared with low‐middle‐income countries and developed countries yet, a review by Yildirim et al. also indicated that the genetic variants of the SARS‐CoV‐2 entry human angiotensin‐converting enzymes ACE2 receptor and related, IFNs, interleukins, TLRs gene, MHC, and ABO gene locus, are critical factors to determine severe COVID‐19.^68^, ^69^

Interestingly, based on global helminth endemic countries, the comparatively low impact of the COVID‐19 disease in tropical and subtropical areas of the world coincides with areas of highly prevalent helminth infections.^68^ Moreover, it is not well known why the severity of COVID‐19 remains lower in most of the resource‐limited nations. Hence, we have attempted to summarize a few studies with hypotheses raised from several researchers as follows.

Bradbury et al.^69^ commentary first described and drew attention to the possible reduction of COVID‐19 severity by helminth co‐infection due to helminth immunomodulation in helminth‐endemic regions. Helminth co‐infection in SARS‐COV‐2 infected patients could derive a parasite‐specific Th2 innate and adaptive immune response with CD4^+^ T cells, eosinophils, IL‐4, IL‐5, and IL‐10 thereby reduce hyperinflammation in patients with severe COVID‐19.^70^ On the contrary, Hays et al.^71^ put their alternative hypothesis stated that helminths may indeed have a mitigating effect based on a theoretical and empirical evidence of the negative impact of helminth infections suggests. Fonte et al.^72^ indicated that helminth coinfection, in conjunction with other factors such as low testing system, age, and genetic background, SARS‐CoV‐2 variant, BCG vaccination, environmental conditions, and endemicity of other infections, could be the possible reasons for low lethality in sub‐Saharan Africa.

Hillyer^73^ has indicated to dedicate towards combating both SARS‐COV‐2 and helminth infections but with an ongoing understanding of their interaction and effects. Here also, other authors have highlighted, not to forget the negative effect of helminths in regions where undernutrition is a dominating concern where it might present a greater hazard in persons at risk for SARS‐CoV‐2 infection.^74^ A recent review also suggested the negative and positive effects of helminth which looks complex and requires exploring the disadvantages and the possible immunomodulatory effects in COVID‐19 together.^75^

Following several hypotheses, a recent study conducted in Ethiopia on COVID‐19 confirmed patients showed that parasite coinfected patients were associated with reduced COVID‐19 severity which suggests that parasite‐driven immunomodulatory response might mute hyperinflammation associated with severe COVID‐19.^76^ In this context, parasite endemicity could be the possible reason for answering why COVID‐19 severity remains lower in those endemic countries. However, not all parasites including protozoa will have immunomodulatory properties.^77^ Besides this, the diversity of helminth species co‐infection resulting in human hosts is such not easily to make conclusions.^70^ While certain helminth infections could reduce the severity of COVID‐19, other helminths at different life cycles can also exacerbate immunopathology. The immune response in acute stage of schistosomiasis is mostly associated with a Th1 type immune response which is dramatically shifted to a Th2 type cytokine expression when the females start to lay eggs.^78^

Research data analyzed from Uganda showed that an inverse correlation between helminth endemic countries and COVID‐19 cases or deaths in the world.^79^ In addition, the direct and indirect effect of helminth in reducing other respiratory viruses could be taken as a lesson, which could potentially reduce pulmonary inflammation induced by COVID‐19. In this context, helminth co‐infections showed reduced COVID‐19 severity. However, there is an urgent need to explore immunological profiles and elucidate the effect of species‐specific parasite immunomodulatory effects on the severity of the SARS‐CoV‐2 infection. To the best of the author's knowledge, no study investigated the implication of helminth infection on the immune response to COVID‐19. Thus, here we tried to show insight based on previous lessons and existing data on the immune response of COVID‐19 related to helminth infection.

## 3 IMPLICATIONS OF HELMINTH INFECTIONS IN COVID‐19 VACCINE RESPONSE: LESSONS FROM OTHER DISEASES

Scientists worldwide have made tremendous efforts to produce vaccines that aimed to protect from COVID‐19. Many vaccine strategies for SARS‐CoV‐2 have demonstrated efficacy in clinical trials, including mRNA encoding of the SARS‐CoV‐2 spike glycoprotein, recombinant spike protein, adenovirus vector expressing the surface glycoprotein, as well as delivery of whole inactivated virus as reviewed from.^80^ As of October 27, 2021, there are 155 vaccine candidates, 485 vaccine trials ongoing, and 42 of these vaccines have entered phase III clinical trials with 23 approved vaccines^81^ and of these about 12 (Table 3) have reported efficacy in the peer‐reviewed literature. Similarly, the vaccination database shows 6.92 billion doses have been administered globally and out of these, 48.9% of the world population has received at least one dose of a COVID‐19 vaccine and only 3.1% of people in low‐income countries have received at least one dose.^82^ Unfortunately, comparing vaccines based on currently available data would be difficult in different study populations under different conditions. Ideally, most COVID‐19 vaccines are designed to elicit strong immune responses, by neutralizing antibodies, against the trimeric SARS‐CoV‐2 spike protein.^83^ Besides this, an effective COVID‐19 vaccine should induce long‐lasting protective immunity with simultaneous involvement of antibody and T cell responses.^84^

**Table 3:** Reported COVID‐19 vaccine efficacy with immunological responses from phase III trials

Vaccine name (Place of production)	Formulations	Reported efficacy	Immunological response
**mRNA‐based vaccines**			
Pfizer – BioNTech (BNT162b2): Germany	Nucleoside‐modified mRNA encoding the viral spike (S) glycoprotein of SARS‐CoV‐2	One dose of vaccine provides 60%–70% protection against symptomatic COVID‐19 and About □80□% protection against hospital admission.^93^ A two‐dose regimen conferred 95% protection in persons with age ≥16 years^94^	Strong IgG response with T_H_1‐skewed T cell immune responses with RBP‐specific CD8^+^ and CD4^+^ T cell expansion. (Increased TNF, IL‐1β and IL‐12p70, but neither IL‐4 nor IL‐5)^95^
mRNA‐1273 vaccine (‎Moderna): USA	Lipid nanoparticle‐based vaccine that encodes, prefusion stabilized, full‐length spike protein of SARS‐CoV‐2^96^	94.1% for symptomatic COVID‐19 wild‐type variants.^97^ 88.1% and 61.3% after first dose; 100% and 96.4% after the second dose against the B.1.1.7 and B.1.351 variants, respectively^98^	A strong CD4^+^ cytokine response involving type 1 helper T cells. Increased TNF α, IL‐2 IFN‐γ responses with minimal IL‐4 and IL‐13.^99^ IL‐15, IFN‐γ, and IP‐10/CXCL10 was associated with vaccine‐induced effective immune response to SARS‐CoV‐2^100^
Cure Vac (CVnCoV): Germany	Unmodified mRNA that encodes full‐length spike surface protein of SARS‐CoV‐2	Inadequate results with only 47% efficacy in phase III trials^101^	Low levels IL‐6, IFNα, while TNF and IL‐1β remained undetectable. no bias toward IFNγ or IL‐4, IL‐5, and IL‐13, indicative of a balanced T_h_1 and T_h_2 response^102^
**Viral vector (non‐replicating) vaccines**			
Oxford/AstraZeneca vaccine (ChAdOx1‐S): UK	Recombinant, replication‐deficient simian adenovirus expressing full‐length SARS‐CoV‐2 spike protein	70% efficacy In adults, >14 days after the 2nd dose.^103^ One dose of vaccine provides 60%–70% protection against symptomatic COVID‐19 and about 80% protection against hospital admission.^93^	Th1‐biased cytokine secretion after vaccination^104^ Increased anti‐SARS‐CoV‐2 spike IgG antibody responses followed by a 12‐week booster dose^105^
Ad26.COV2.S (Janssen/Johnson & Johnson): USA	Adenovirus serotype 26 (Ad26) vector expressing a stabilized pre‐fusion stabilized spike protein	67% efficacy against moderate to severe COVID‐19 after a single dose and 85% against the risk of developing severe COVID‐19^106^	No IL‐4 responses were observed, indicating a T_H_1‐biased cellular immune response. IFN‐γ responses correlated with Spike‐specific binding antibody titers^109^, ^110^
CanSino Biologics ‐ Ad5‐nCoV: China	Recombinant adenovirus type 5 vector expressing full‐length Spike protein	65.28% of symptomatic cases and 90.07% of severe diseases after a single dose interim analysis^109^	The specific memory CD4^+^ T cells secreted IFN‐γ and IL‐2 but not IL‐4 and IL‐13 in all groups at Day 14 after the initial vaccination; similarly, memory CD8^+^ T cells secreted mainly IFN‐γ and low concentrations of IL‐2. Induced strong IgG and neutralizing antibody responses^110^
Gam‐COVID‐Vac (Sputnik V): Russia	Recombinant adenovirus type 26 (rAd26) vector and a recombinant adenovirus type 5 (rAd5) vector, both carrying the full‐length spike protein^111^	78.6% efficacy for preventing infections, 87.6% and 84.8% efficacy for reducing hospitalization and death respectively in population aged 60–79^112^	Increased antigen‐specific T‐cell responses and interferon‐γ concentration with a SARS‐CoV‐2 neutralization antibody^111^
		91.6% from 21 days after the first dose of vaccine in phase III trial^111^	
**Inactivated vaccines**			
Bharat Biotech (Covaxin): India	SARS‐CoV‐2 grown in Vero cells, soaked in beta‐propiolactone mixed with the aluminum‐based adjuvant Alhydroxiquim‐	78% in phase III trials	Th1 skewed profile (higher IFN‐γ and TNF) with minimal IL‐5 and IL‐13^113^
Sinovac – Corona Vac: China	SARS‐CoV‐2 grown in Vero cells, soaked in beta‐propiolactone, and adsorbed onto aluminum hydroxide	Efficacy trials have announced efficacies (for the same product) of 50%, 65%, 78%, and 91%^114^	Not reported
Sino pharm (BBIBP‐CorV): China	β‐propiolactone‐inactivated HB02 strain of SARS‐CoV‐2 grown in Vero cells	79% against COVID‐19 from Phase III trials	No notable change of cytokine subsets in phase 1/2 trial^115^
**Protein subunit**			
Novavax (NVX‐CoV2373): USA	A recombinant nanoparticle full‐length spike glycoprotein of the prototype strain plus Matrix‐M adjuvant ^116^	Has differential protective immunity against the parental strain, B.1.1.7, and B.1.351 in clinical trials 96%, 86%, and 60%, respectively^117^	CD4^+^ T cell responses present by 7 days after second dose, based on IFNγ, IL‐2 and TNF production in response to S protein stimulation, with a strong bias toward a TH1 cell phenotype; minimal Th2 cell responses (as measured by IL‐5 and IL‐13)^118^
Zifivax (ZF2001): China	RBD of the S protein at C‐terminal domain of S1 subunit	81.76% against COVID‐19 from Phase III trials	Elicited moderate levels of both Th1 (IFNγ and IL‐2) and Th2 (IL‐4 and IL‐5) cytokine production after the immunizations in phase 1 trial^119^

Abbreviations: BBIBP‐CORV, Beijing Bio‐Institute of Biological Products Coronavirus Vaccine; IFNγ, interferon‐γ; IgG, Immunoglobulin G; IL, interleukin‐2; rAd5, recombinant adenovirus type 5; RBP, Receptor binding protein; S, Spike; CD, cluster of differentiation; SARS‐CoV‐2, severe acute respiratory syndrome coronavirus 2; TH1 cell, T helper 1 cell; TNF, tumor necrosis factor.

Despite several factors that could reduce the efficacy of COVID‐19 vaccines, here the focus of our review highlights the effect of helminth on COVID‐19 vaccine efficacy particularly in helminth endemic countries that need extensive research based on the following baseline data from other diseases.

Several studies have been shown helminth co‐infection and anthelminthic therapy could reduce and enhance the efficacy of a vaccine against several pathogens respectively. *Litomosoides sigmodontis* nematode infected BALB/c mice model has been shown suppression of the humoral response to thymus‐dependent vaccination, thereby the numbers of antigen‐specific B cells, as well as Th2‐associated IgG1 and TH1‐associated IgG2 responses were suppressed.^85^ Helminth infection impaired the immunogenicity of a *Plasmodium falciparum* DNA vaccine.^86^ On contrary, in mice model to malaria transmission‐blocking vaccine *P. falciparum* (pfs230D1‐EPA/Alhydrogel®), Chronic helminth infection has induced a marked increase in systemic Th2 and regulatory cytokine levels in but could not able to alter vaccine specific‐antibody level immune response.^87^ Reduced antibody response to the live attenuated oral cholera vaccine CVD 103‐HgR has been observed in children treated with Albendazole for ascariasis.^88^ Moreover, reduced cellular and humoral responses have been observed in humans for tetanus toxoid with concurrent *Wuchereria bancrofti* infection.^89^

In a trial done in the USA for the efficacy of pneumococcal vaccine, mice that have been vaccinated with either commercial conjugate or purified polysaccharide vaccines had impaired antibody responses if they were chronically infected with *Taenia crassiceps*. This translated to increased susceptibility to pneumococcal pneumonia and high mortality compared to helminth‐negative vaccinated animals, which were fully protected from disease and death. Antibodies taken from Taenia‐infected vaccinated mice were unable to effectively opsonize *S. pneumoniae* for killing by alveolar macrophages and did not protect against pneumococcal challenge when adoptively transferred into naïve animals.^90^

A study done in Uganda has been shown that helminths are known to have implications in response to immunization of BCG, and on the incidence of infection and disease. Clinical trials done in Ethiopia to explore the effect of deworming on human T‐cell response to mycobacterial antigen showed anthelminthic therapy improved mycobacterial antigen (PPD) specific cellular responses compared with the placebo group.^91^ On the other hand, studies of clinical trials showed BCG vaccination had immunomodulatory properties that could protect against respiratory infections. It has been hypothesized that Bacillus Calmette–Guerin (BCG) vaccination might reduce the severity of COVID‐19 by inducing trained immunity leads to epigenetically trained populations of monocytes and/or natural killer cells, which most likely reside in the bone marrow.^65^

Helminths reduce Th1 and Th17‐induced antiviral activity and vaccination efficacy.^75^ Here, from findings of previous studies on the effect of helminth in reducing vaccine efficacy, several approved vaccines of COVID‐19 could have various efficacies in different populations. Thus, considering studies that assess the response of vaccines in a variety of geographical and demographic areas may give worthful data and it will enhance the efforts of the world to end the pandemic.

Importantly, lessons from other respiratory viral infections have been also shown that helminth co‐infection was reduced the efficacy of the vaccine. Hartmann et al.^92^ demonstrated in *L. sigmodontis* infected mice accompanied by a sustained and systemic expansion of sustained expansion of CD49b, lymphocyte activation gene‐3 (LAG‐3), Treg1 cells, and IL10 with reduced neutralizing antibody. This was associated with prolonged suppression of vaccine efficacy even after clearance of their acute helminth infection which they have been suggested helminth endemic areas might not benefit from vaccinations from seasonal influenza (H1N1).^92^ In this context, one can hypothesize; people who are infected with helminth or had chronic helminth infections especially those people living in helminth endemic countries could have a reduced COVID‐19 vaccine response.

## 4 CONCLUSIONS AND FUTURE PERSPECTIVES

Several authors have got attention to the immunomodulatory effects of helminth in COVID‐19 patients. It is crucial exploring more about the immune signature of helminth co‐infection and other confounding factors with COVID‐19 severity. Identifying immunological mechanisms and immunomodulatory components could bring new insights into the immunological and molecular mechanisms, which have a dual benefit to enable the current efforts for mitigating COVID‐19 and enhancing the efficacy of current vaccines. The effect of helminth on vaccine efficacy in different conditions remains unclear and relatively little information is available. It is essential to balance the negative impact of helminth infections in resource‐limited countries and potential immunomodulatory effects in COVID‐19 patients. In general, we conclude, evidence‐based data are urgently needed to identify the immunomodulatory effect of helminth‐ in COVID‐19 patients that could have an impact on the clinical illness associated with SARS‐CoV‐2 infections in humans. Moreover, information gleaned from such studies on COVID‐19 vaccine efficacy in helminth endemic countries will directly influence recommendations regarding whether deworming interventions for at risk communities in COVID‐19 patients.

## CONFLICT OF INTERESTS

The authors declare that there are no conflict of interests.

## AUTHOR CONTRIBUTIONS

Yibeltal Akelew wrote the manuscript draft. Henok Andualem, Endris Ebrahim, Aytenew Atnaf, and Wasihun Hailemichael contributed to the gathering of data, draft reviewing, and editing of the manuscript. All authors revised the manuscript and approved the final version of the manuscript before submission.

## Data Availability

Data sharing not applicable to this article as no data sets were generated or analyzed during the current study.
